# Nest Sanitation as the Evolutionary Background for Egg Ejection Behaviour and the Role of Motivation for Object Removal

**DOI:** 10.1371/journal.pone.0078771

**Published:** 2013-11-06

**Authors:** Miroslav Poláček, Matteo Griggio, Michaela Bartíková, Herbert Hoi

**Affiliations:** 1 Institute of Zoology, Slovak Academy of Sciences, Bratislava, Slovakia; 2 Konrad Lorenz Institute of Ethology, Department of Integrative Biology and Evolution, University of Veterinary Medicine, Vienna, Austria; Hungarian Academy of Sciences, Hungary

## Abstract

Higher interclutch colour variation can evolve under the pressure of brood parasitism to increase the detection of parasitic eggs. Nest sanitation could be a prerequisite for the evolution of anti-parasite defence in terms of egg ejection. In this respect, we used nest sanitation behaviour as a tool to identify: i) motivation and its underlying function and, ii) which features provoke ejection behaviour. Therefore, we experimentally tested whether size, colour or shape may influence ejection behaviour using artificial flat objects. We found a high interclutch variation in egg colouration and egg size in our tree sparrow (*Passer montanus*) population. Using colour and size we were in fact able to predict clutch affiliation for each egg. Our experiments further revealed the existence of direct anti-parasite behaviours and birds are able to recognise conspecific eggs, since only experimentally-deposited eggs have been removed. Moreover, experiments with different objects revealed that the motivation of tree sparrows to remove experimental objects from their nests was highest during egg laying for objects of varying size, most likely because of parasitism risk at this breeding stage. In contrary, motivation to remove white objects and objects with edges was higher during incubation stage as behavioural patterns connected to hatching started to emerge. The fact that rejection rate of our flat objects was higher than real egg ejection, suggests that egg ejection in tree sparrows and probably more general in small passerines, to be limited by elevated costs to eject eggs with their beaks. The presence of anti-parasite behaviour supports our suggestion that brood parasitism causes variation in egg features, as we have found that tree sparrows can recognise and reject conspecific eggs in their clutch. In conclusion, in tree sparrows it seems that nest sanitation plays a key role in the evolution of the removal of parasitic eggs.

## Introduction

Brood parasitism is a reproductive tactic observed in many bird species where eggs are left under the parental care of the host parents [1]–[3]. There are obvious advantages for the parasites, namely they save energy by not paying for rearing their young, and consequently have free energy to produce more eggs [4], [5] and by distributing eggs to different host nests they spread the risk for their offspring, as the likelihood that at least one offspring survives, increases [6]. In contrast, brood parasitism is detrimental for the host and its own chicks and, in the best scenario, the host simply wastes energy feeding unrelated offspring [2], [3].

Hosts consequently develop defence strategies to avoid egg dumping attempts. Regarding defence strategies of host individuals, the most commonly used are defence behaviours based on egg recognition [7]–[9] and counting eggs [10]. Some species recognise and eject conspecific eggs or eggs of brood parasites and in this way, significantly save fitness and energy costs. Hosts may also move parasitic eggs to outer incubation positions [10], bury them in the nest material [11] or abandon the clutch and build a new nest [12]–[14]. Theoretical models suggest that, in general, egg ejection should be the superior anti-parasite strategy to nest abandonment, but increasing parasitism rates and increasing fitness values of host eggs may influence fitness payoffs in favour of nest abandonment [15].

One key feature for any anti-parasite action of the host however, is to discriminate parasitic eggs from its own eggs. In line with this, variation in egg colouration, in particular interclutch variation has been suggested to enhance the discrimination of own from parasitic eggs [16], [17].

The rejection rate of parasitic eggs is frequently much lower than parasitism [16], [18]. In this context, some host species do better and some seem to be insensitive against any parasitism attempt even when differences are very obvious. An imperfect host recognition system is used to explain this puzzling phenomenon [19]–[21]. One aspect which seems to us largely ignored to understand whether or not an individual removes a foreign object is the role of host motivation and the driving force behind. Soler and colleagues [22] suggested that egg rejection is a stepwise process with each step being more costly. Motivation plays a key role in host's decision to accept or continue to the next step. Motivation to reject a parasitic egg might increase with a higher risk of parasitism [12]. We would like to suggest here a mechanistic approach. One possibility could be that motivation to remove a foreign object from the nest increases with its dissimilarity to known objects (own eggs). In marsh warblers (*Acrocephalus palustris*) motivation to reject was higher when the non-mimetic egg was placed in the nest, represented by higher pecking rate and strength [23]. Alternatively birds might be highly motivated but are prevented to do so, because they are incapable e.g. to grasp the egg with the beak or to discriminate own from conspecific eggs without high certainty. Furthermore the motivation for egg ejection might have an evolutionary background. In a recent review, Guigueno and Sealy [24] suggested that nest sanitation plays a role in the evolution of the removal of parasitic eggs and, hence, should be a prerequisite for the evolution of anti-parasite defence in terms of egg ejection [25]–[27]. The existence of nest sanitation hence might be of great impact for the existence of egg ejection behaviour or drive the motivation to eject any object including a parasitic egg from the nest. Having this in mind the motivation may also change with time depending on the needs. If the driving force is mainly nest sanitation, one can predict that motivation for object ejection may increase with the time and peak when nestlings appear, if anti-parasite behaviour is the driving force one may predict a peak in ejection behaviour during the egg laying period.

To investigate if nest sanitation plays a role in the evolution of the removal of parasitic eggs we used the nest sanitation behaviour as a tool to identify: i) motivation and its underlying function and, ii) which features may provoke ejection behaviour. Since motivation may be hidden by the ability of an individual to recognize and remove real eggs (see earlier), we used flat objects in this study. Flat models of eggs are obviously easier to eject than real eggs [24].

To better understand egg rejection, it is also important to know which egg features play a role in parasitic egg recognition. Most studies focused on different aspects of eggshell colouration suggesting that the acceptance of a parasitic egg depends on how well it matches host egg colour and patterning [7], [28]. Additionally, it was shown that multiple cues of colour and pattern are used simultaneously [29] and the phenotype of the blunt pole is critical in egg discrimination [30]. Along with colouration, hosts might use also size differences to identify parasitic eggs [12], [21], [31], [32]. Non-egg-shaped objects, with edges and/or flat surfaces, are removed at a higher rate than egg-shaped objects [24]. Great reed warblers (*Acrocephalus arundinaceus*), a frequent host of the common cuckoo (*Cuculus canorus*), removed rounded egg models with two blunt poles at a higher rate than normal shaped eggs, and the model with two sharp poles had the lowest rejection rate. Thus, even object shape might be subject of a coevolutionary arms race between the host and the parasitic bird [33].

In our study, we focused on conspecific brood parasitism in the cavity breeding tree sparrow (*Passer montanus*). This species is an ideal model species because observational data of our group suggest strong egg size and colour variation between clutches. Cuckoo parasitism and nest predation can be neglected as selective pressures for egg shell colouration in cavity breeders and is even more unlikely to explain high interclutch variation in egg shell colouration. Furthermore tree sparrows have in fact a very flexible mating system varying from polygyny over monogamy to and in particular polyandry [34], where egg dumping may be part of female reproductive strategy. Furthermore, Soler and Møller [35] observed high interclutch colour variation in the closely related house sparrow (*Passer domesticus*) and this species seems to react to experimental brood parasitism with egg ejection [36]. However, several recent studies found only very low rates of conspecific brood parasitism in this species [37]–[39]. Finally conspecific brood parasitism was also reported in tree sparrows [14] and they also seem to be able to remove eggs by puncture ejection [40]. However, as far as we know, there is no detailed investigation exploring this phenomenon.

Therefore, in the current study we first explore the possibility for conspecific brood parasitism and anti-parasite behaviour in our study species. We look into the degree of interclutch variation in egg features describing size, colour and shape and the frequency of several anti-parasite behaviours including egg ejection, nest desertion, or behaviours which may put those eggs in an unfavourable position (e.g. egg burial). One would expect high interclutch variability and high efficiency of anti-parasite behaviour when there is a high pressure of conspecific brood parasitism. Second, we wanted to know whether and to what extend tree sparrows are able to recognise conspecific parasitic eggs and which features provoke egg ejection behaviour. In line with this we experimentally tested whether size, colour or shape may influence ejection behaviour using artificial flat objects. Third, to study the motivation behind egg ejection behaviour we examined changes in this behaviour between egg laying and incubation as a reaction to different selective pressures acting on these distinct breeding stages. We would predict ejection behaviour due to the risk of egg dumping to be more important during the egg laying stage [41]–[43], whereas during incubation, ejection behaviour might be more likely due to nest sanitation. To answer this question, we experimentally tested reaction to the same set of flat objects more or less similar to real eggs during egg laying and incubation. In line with this we also explored whether nest sanitation behaviour may be also sex-specific. Indeed, there is almost no information about differences between sexes in response to experimental objects [24]. Fourth, we compare and discuss the results derived by artificial and natural observed ejection behaviour.

## Methods

### Ethics statement

The study was done under the permission of the Nature conservation department of Lower Austria (RU5-BE-7/010-2011) and the experiments were proofed by the ethical commission of the Austrian Ministry of Sciences (BMWF-68.205/0245-II/3b/2012).

We always used eggs coming from deserted and incomplete clutches for parasitic eggs in our egg recognition experiment. In this way we could avoid that the parasitic egg contained a developed embryo which would have been killed by our procedure.

The study was done on private vineyards where we had the permission to work with our birds.

### Study species and fieldwork

The research was performed in an agricultural landscape of Lower Austria, in the vicinity of the village of Feuersbrunn (48°26′ N, 15°47′ E). Our study population was breeding in nest boxes installed in vineyards (288 in 2010, 296 in 2011), fence posts and trees in an apricot orchard (36 in 2010, 43 in 2011). Pairs usually had three broods per year with an average of 5.096±0.049 (mean ± SE) eggs. Nest building started in the first week of April. Hence, from the beginning of April, all nest boxes were checked weekly. Occupied nest boxes (with nest material) were then checked daily to determine laying order.

Digital pictures of clutches were taken after the beginning of incubation. To keep light conditions as standard as possible, all pictures were taken inside a wooden box through a camera slot at the top fitting the camera Fuji Finepix S200-EXR and the ring flash. Moreover, each clutch was photographed with a white standard (Top Sensor Systems WS-2). Pictures were saved in RAW format and then processed in Adobe Photoshop. Firstly, the colour of each picture was adjusted according to the white standard. CIE Lab colour space was used for colour measurement. Four measurements were taken per egg at four randomly chosen places that were evenly distributed on the picture of each egg. The average of these measurements was further used to analyse Lightness (L*) which represents the achromatic properties of an object, ranging from 0 (black) to 100 (white). Beside colour measurements, we determined 6 other egg shell colour characteristics like categories for spot size (from 1 – small spots to 4 – big spots), spot density (from 1 – clearly separated spots to 5 – impossible to recognise individual spots), spot distribution (from 1 – equal distribution along longitudinal axis to 4 – over 90% on a blunt pole), shell glossiness (from 1 – dull to 3 – glossy), pigment intensity (from 1 – light red to 4 – dark brown) and contrast between background and spots (from 1 – hard to recognise borders to 3 – clear contrast). Categories were determined on all egg pictures and were evaluated by one observer (MP) to reduce variance produced by estimates of different observers.

Regarding egg size and shape, we measured the width and length of eggs and derived egg volume from these measurements according to Hoyt [44]. As a basic shape parameter, we used the ratio of egg width to length.

### Experiment I: Egg recognition

We added one conspecific egg to the nest after females laid their second egg. In this way we tested 19 nests in the 2010 breeding season. Experimental clutches were monitored on a daily basis and the experimental egg was considered accepted when host chicks started to hatch. Experimental parasitic eggs originated from the same population and were collected from nest boxes of individuals of unknown identity. Experimental eggs were randomly taken from an available stock. To avoid eggs with already developed embryos, the eggs used as parasitic eggs came from deserted incomplete clutches. Eggs were fresh because they were collected as soon as desertion was detected and stored in the fridge at 4°C. Accordingly, when collected, age of experimental eggs was between 4 to 7 days and usually 7 to 12 days during the experiment. One experimental egg was used three times and accepted in all cases.

### Experiment II: Temporal changes and characteristics inducing egg removal

**Figure 1:**
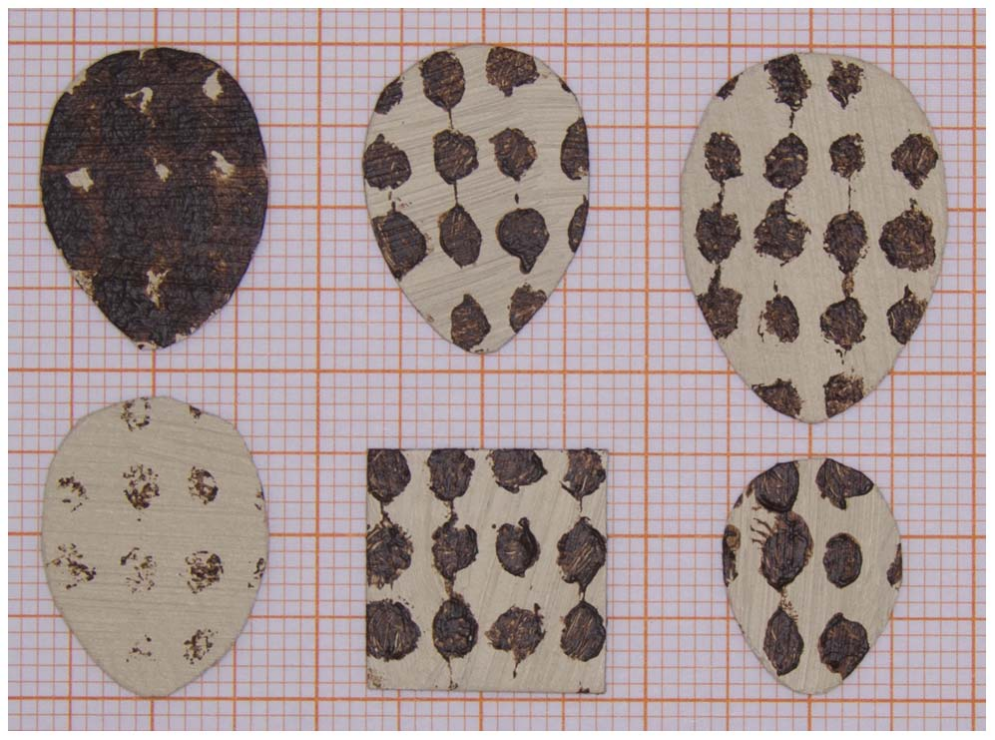
Experimental object design. Experimental objects used in the experiment II. First row from left is “dark egg”, “normal egg” and “big egg”. Second row “white egg”, “square” and “small egg”.

**Figure 2:**
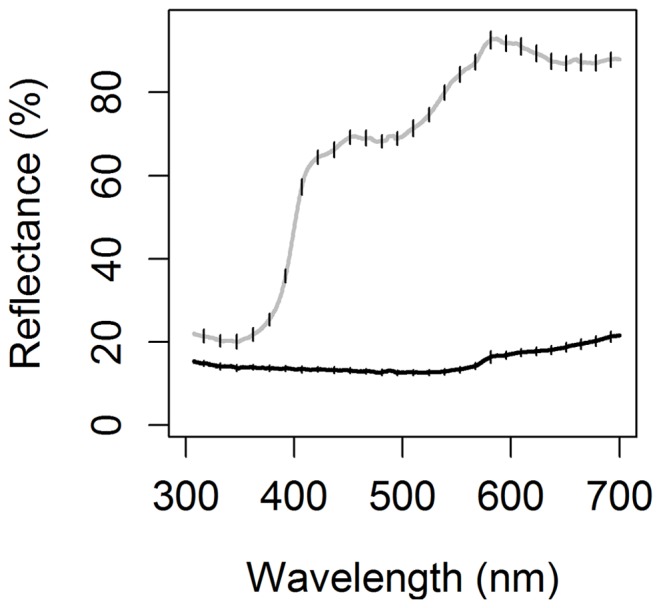
Colour spectra of experimental objects. Reflectance (%, mean ± SE) spectra of two acrylic paints, dark brown (black curve) and light brown (grey curve) used for experimental objects.

To examine motivation behind ejection behaviour in relation to similarity we separated the effects of size, colour and shape on egg ejection behaviour and, created six experimental object types (see Figure 1). These objects were flat, originated of the same material (cardboard and acrylic paints). All objects were derived from a basic object, referred to here as a “normal egg”, which had an egg contour (mean ± SE: length 19.41±0.08 mm; width 14.30±0.07 mm; mass 0.101±0.001 g). This reflects the egg dimensions in our study population (mean ± SE: egg length 19.08±0.036 mm; egg width 14.05±0.021 mm; based on 592 eggs, 117 nests) in 2010. The background colour of the “normal egg” was light brown with dark brown spots evenly imprinted over the surface. For size, we prepared a “small egg” (mean ± SE: length 14.98±0.08 mm; width 11.39±0.08 mm; mass 0.059±0.001 g) which was smaller than a “normal egg” and a “big egg” (mean ± SE: length 23.33±0.08 mm; width 16.51±0.07 mm; mass 0.142±0.001 g) which was bigger than the “normal egg”. To investigate the role of colour, we used objects of the same dimension as the “normal egg” but the “dark egg” had large spots imprinted to the level that they became connected. Therefore, the “dark egg” was almost completely dark brown. The “white egg”, had spots lightly painted. For shape, we used an object with edges as “square” (mean ± SE: length 15.22±0.04 mm; mass 0.106±0.001 g) and the “normal egg” represented the round control object. The “square” and “normal egg” had the same surface and colour. Reflectance curve for two paints used on experimental objects (see Figure 2) was obtained by an Ocean Optics S2000 Spectrometer. A fibre-optic measuring probe (Ocean Optics) was used to transfer the light from a deuterium tungsten halogen light source (Ocean Optics DH-2000-BAL) to the measured surface and to pass the light reflected back to the spectrometer. The probe was held at a 90° angle to the measured surface and ambient light was excluded with a black tube that held the probe tip at 4 mm distance from the surface. Five consecutive measurements were taken, lifting and replacing the probe each time, and averaged [45].

For each test we used only one model object which may raise some problem regarding pseudoreplication. However, all model objects originated from the same material with only one character significantly different (size, shape or colour). Thus we assumed that no other than the manipulated characteristics will have important consequences for the outcome of our results. From the day when the first egg appeared in the nest we assigned nest boxes randomly to a treatment group. Experiments were performed in April and May 2011.

Regarding changes in the motivation we tested 30 nest boxes during egg laying (egg laying + 2 days after the last day of laying) and 30 during incubation (day 6 of incubation ± 2 days). Day 0 of incubation was the day when the last egg was laid. Day 6 was chosen because it is approximately in the middle of the incubation stage [34]. Each nest was tested only in one stage, e.g. egg laying or incubation, but twice with different objects. There was always a minimum of one day between experiments of each nest. Tested objects were placed in the nest cup on the side opposite to the entrance hole. After adding the experimental object, we observed the nest box either directly via telescope or by using a digital video camera. Observations lasted for 60 min.

### Statistical analyses

A stepwise discriminant function analysis was used to determine differences in egg size and colour features between females (clutches). In these analyses, only clutches with known female identity from the breeding season of 2010 were used. The grouping variable was female identity and 11 independent variables were used altogether representing egg size (length, width, shape, volume) and egg colour (lightness, spot size, spot density, spot distribution, glossiness, pigment intensity, spot-background contrast). A stepwise discriminant function analysis was further used for size and egg shell colouration variables separately to determine whether any set of variables, related to size or colour alone, might be able to discriminate between clutches of different females. The SPSS statistical program (version 20) was used.

To determine motivation we used the reaction time until objects were removed in relation to different size, colour and shape. More precisely, the motivational (dependent) variable was latency to rejection, which was the time from when an individual entered the nest box for the first time until it appeared again in the hole with the experimental object in its bill. The data were analysed by accelerated failure time models in the R statistical program (R Development Core Team, 2012), package ‘survival’ [46]. In cases where the object was not removed during the observation period (one hour), we used these data in the analyses as right censored. Because of the presence of such censored data we used the accelerated failure time model. Independent variables were object, breeding stage (egg laying and incubation) and their interaction. The nest box identity was included as a repeated factor in the analyses of size and colour; for shape we used each nest box only once. The final sample size for this analysis is reduced for “small egg” during egg laying to 7, and for “big egg” and “dark egg” during incubation to 9 because we missed the point of first entrance of the bird to the nest box. Additionally size, colour and shape were also tested separately with the “normal egg” as a control.

One additional analysis was performed to identify differences in latency to rejection depending on the original clutch colour in relation to the colour of the tested object. For this reason, difference in lightness between clutch and object was added to model. We performed this analysis separately because the data for original clutch colour are available only for a smaller sample size.

In addition, we were interested in the immediate reaction to an object in the nest. Rejection rate in the first two minutes was calculated and these data were then compared by a 2×3 Freeman-Halton exact contingency test [47].

## Results

### Is there interclutch variability in egg colour and size?

A discriminant function analyses revealed that egg features significantly differ between clutches of different females based on several variables (*F* = 11.04, *df* = 9.132, *P*<0.001). According to the analyses, egg length, width, shape, volume, lightness, spot size, spot distribution, shell glossiness and pigment intensity entered the model and together explained 97.3% of the variation. In fact, based on the model generated, 88.41% of all eggs could be correctly assigned to the clutch from which they were collected. However, there were still 19 (11.59%) mismatching eggs from 15 nests. Eight of these eggs were the last in the laying order, five were the first and six of them were in between. In one of these clutches with mismatching eggs, laying was interrupted for one day after the second and again after the third egg. In all other clutches we did not detect any abnormalities in the laying sequence.

To determine whether size or colour is more important to discriminate mismatching eggs, stepwise discriminant function analyses were performed separately for size and colour variables. Colour (*F* = 11.604, *df* = 6.132, *P* = 0.0001) was deemed to be much more important for discriminating between clutches of different females, explaining 95.4% of the variation compared to size (*F* = 9.814, *df* = 4.132, *P* = 0.0008), which explained 91.3% of variation. For size, 57.37% of eggs were mismatched, including at least one egg in each clutch. In contrast, only 24.39% of the eggs of 17 nests were mismatched when considering only colour.

### Experiment I: Are tree sparrows able to recognise parasitic eggs and do they show any anti-parasite behaviour?

In two out of 19 (10.5%) cases, where an extra egg was added, the experimental egg was removed. In the other three cases (15.8%) the nest was deserted immediately after experimental parasitism. Furthermore, one experimental clutch was deserted during incubation and a new nest was built on top of the experimental clutch in one other case. Thus, in 7 (36.8%) out of 19 clutches, where an experimental egg was added, the host pair revealed an anti-parasite behaviour. However there was still a high proportion of clutches (63.2%), where the birds continued incubating the clutch with an extra egg. Nest desertion in experimental nests was 26.32% (5 nests). In comparison, desertion during egg laying and incubation in nests which were not used in this experiment was 11.54% (first brood in 2010, *N* = 52). Thus considering this rate as the “expected” rate of nest desertion under the null hypothesis of no recognition, a *X* ^2^-test revealed nest desertion to be significantly higher in parasitized nests (*X* ^2^ = 3.96, *df* = 1, *P*<0.038).

Furthermore, none of the mismatching eggs were put into an unfavourable position (e.g. nest rim, below the other eggs or moved more frequently) and all accepted parasitic eggs were warm during nest inspections.

### Experiment II: How important are colour, size and shape for eliciting ejection behaviour?

Reactions to the tested flat experimental objects were strong. Within 1 hour, 81.7% of the objects were rejected. The difference in rejection rate between egg laying (75%) and incubation (88.3%) was almost significant (binomial test: *z* = 1.89, *P* = 0.059). Objects were removed by both sexes but significantly more often by the females. In 25 cases where the sex was determined, we found 21 females removed experimental objects out of 22 cases (95.5%) when female had opportunity to remove it, entered nest box with experimental object. Males had 11 opportunities and removed an object in 4 cases (36.4%) (binomial test: *z* = 3.73, *P* = 0.0002, egg laying and incubation experiments pooled).

### Removal behaviour in relation to object size

**Figure 3:**
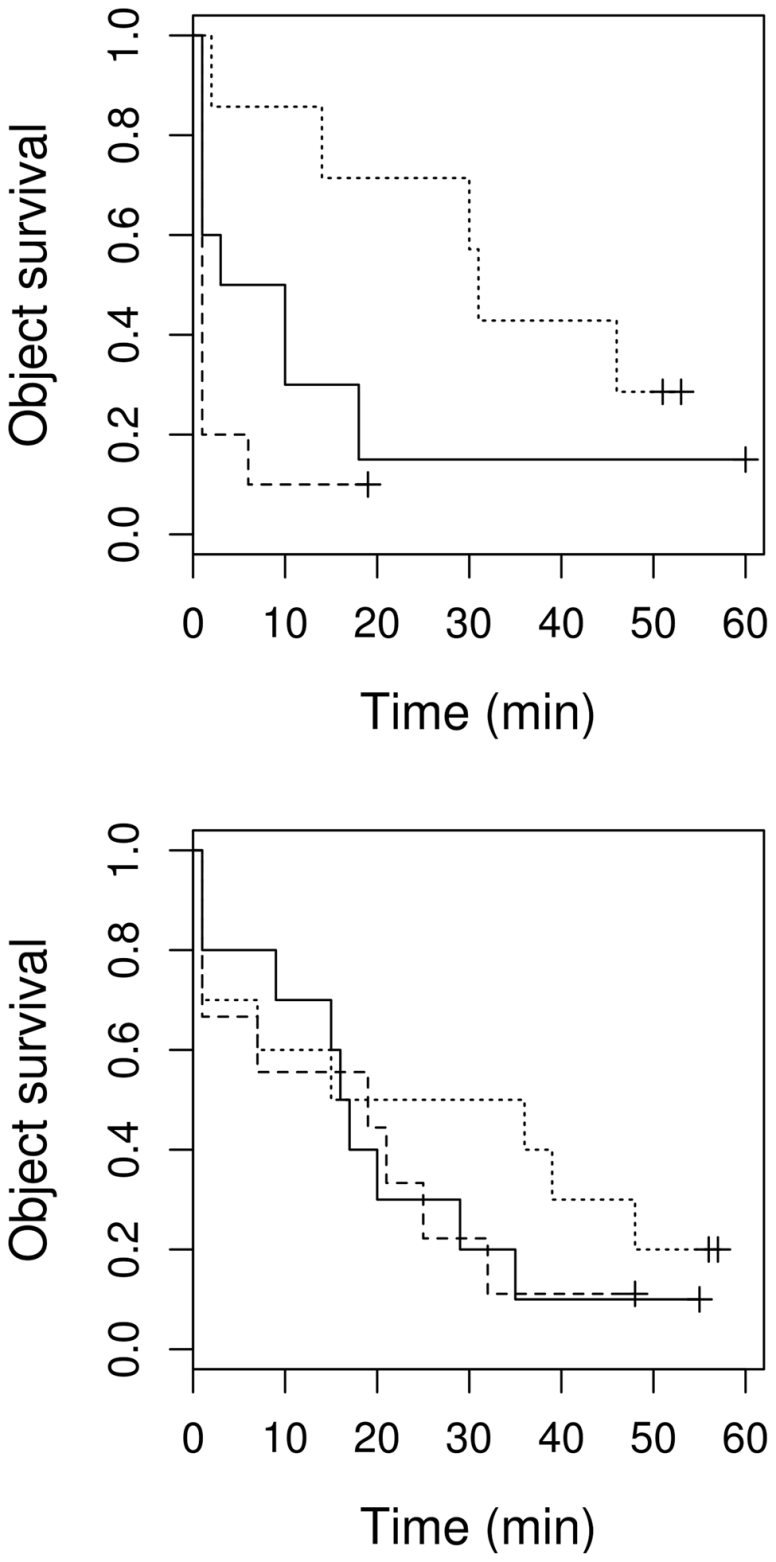
Size dependent object survival in the nest. Survival curves of experimental objects varying in size (dashed line - “big egg”, solid line - “normal egg”, dotted line - “small egg”) in a nest during egg laying (upper) and incubation (lower). Vertical tick-marks indicate censored data.

Object size had a significant effect on latency to rejection (*X* ^2^ = 7.16, *df* = 2, *P* = 0.028) and the interaction between object size and breeding stage was also strongly significant (*P*<0.001). During egg laying, “big eggs” were removed faster than “small eggs” and “normal sized eggs” were removed at an intermediate rate (Figure 3). During incubation, however, there was no obvious difference between objects of different sizes (Figure 3), and there was no significant difference in the removal pattern between the egg laying and incubation stages (*X* ^2^ = 2.54, *df* = 1, *P* = 0.111).

**Figure 4:**
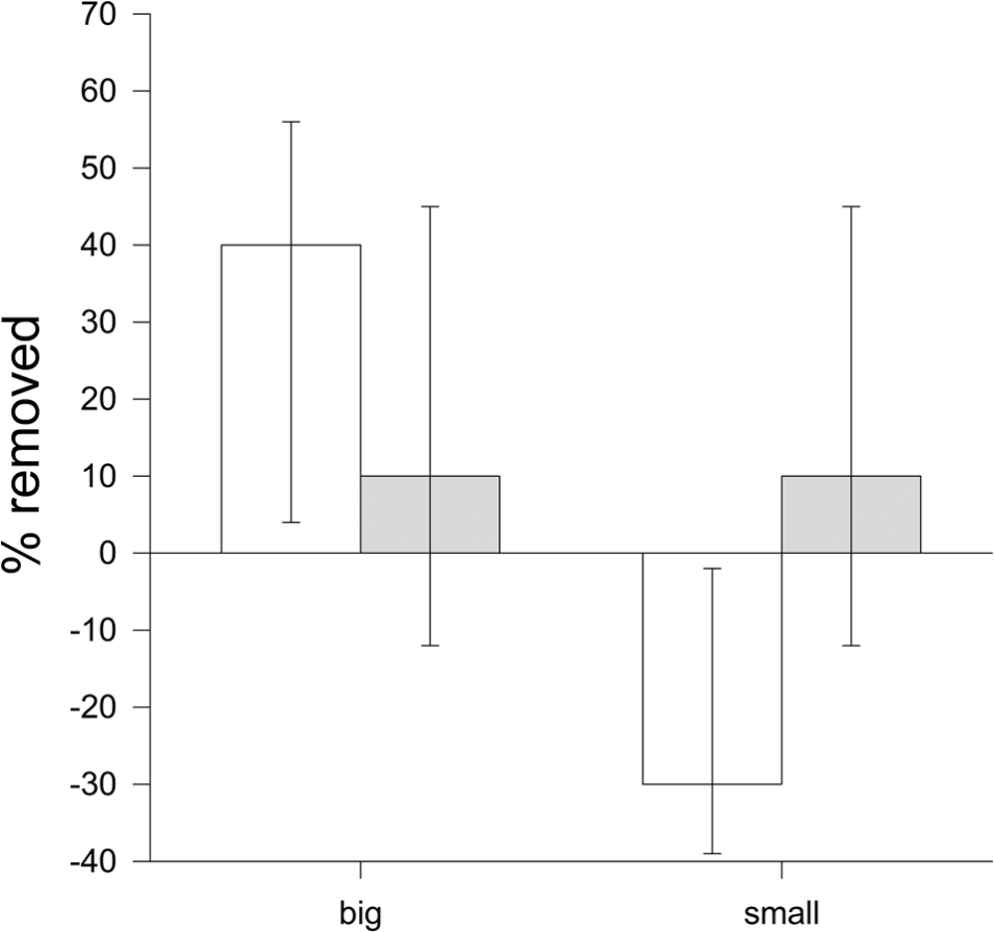
Immediate rejection in relation to object size. Mean ± SE proportion of objects rejected in the first 2 min for objects varying in size during egg laying (white bars) and incubation (grey bars). Results are expressed as the difference in rejection rate in comparison to the “normal egg” (normal egg represents the zero line).

Examining only the removal rates immediately after the first 2 min, we found a significant effect of size during the egg laying stage (*df* = 3, *z* = 2.8, *P* = 0.0046, Figure 4). During egg laying, significantly more egg-shaped objects were removed when they were bigger than normal (*z* = 2.6, *P* = 0.009) and significantly less egg shape objects were removed when they were smaller than normal (*z* = 3.22, *P* = 0.001). However, during incubation, no size-dependent variation could be detected (*P*>0.4).

### Removal behaviour in relation to object colour

The mismatch in colouration between the experimental object and the original clutch did not seem to influence latency to rejection (*X* ^2^ = 0.63, *df* = 1, *N* = 51, *P* = 0.428).

**Figure 5:**
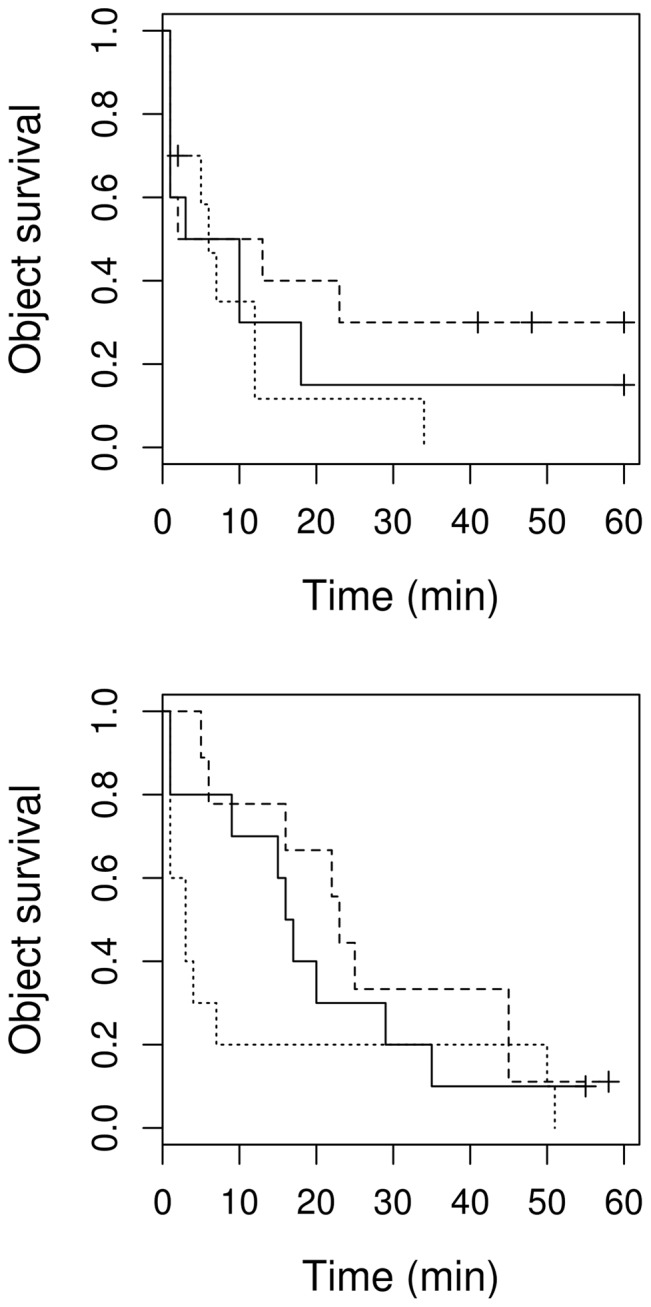
Colour dependent object survival in the nest. Survival curves of experimental objects varying in colour (dashed line - “dark egg”, solid line - “normal egg”, dotted line - “white egg”) in a nest, during egg laying (upper) and incubation (lower). Vertical tick-marks indicate censored data.

**Figure 6:**
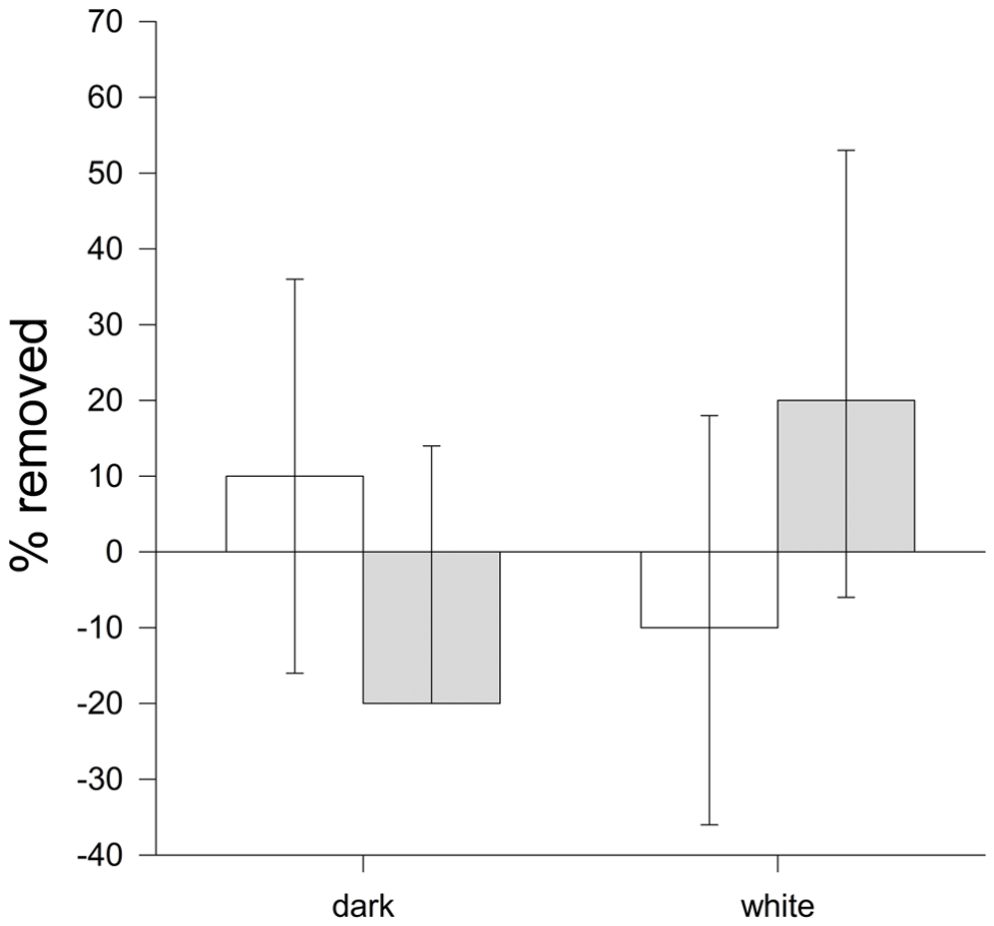
Immediate rejection in relation to object colour. Mean ± SE proportion of objects rejected in the first two min for objects varying in colour during egg laying (white bars) and incubation (grey bars). Results are expressed as the difference in rejection rate in comparison to the “normal egg” (normal egg represents the zero line).

The latency to reject objects with different colours, however, differed significantly (*X* ^2^ = 6.01, *df* = 2, *P* = 0.049). There was no significant difference between egg laying and incubation (*X* ^2^ = 0.50, *df* = 1, *P* = 0.48), but the interaction between object and breeding stage was strongly significant (*P*<0.001). In line with the fact that birds may perceive white eggs as faecal sacs, the removal of “white eggs” was faster than that of “dark eggs” (Figure 5). Examining only the removal rates immediately after the first 2 min in relation to colour variation, we found a significant effect of colour during the incubation stage (*z* = 2.2, *df* = 3, *P* = 0.025) but not the egg laying stage (*P*>0.5; Figure 6). In particular, significantly fewer dark egg-shaped objects were removed in comparison to normal coloured ones (*z* = 2.07, *P* = 0.031), while the opposite seems to be true for white-coloured objects, although this was not statistically significant (*P*>0.23).

### Removal behaviour in relation to object shape

**Figure 7:**
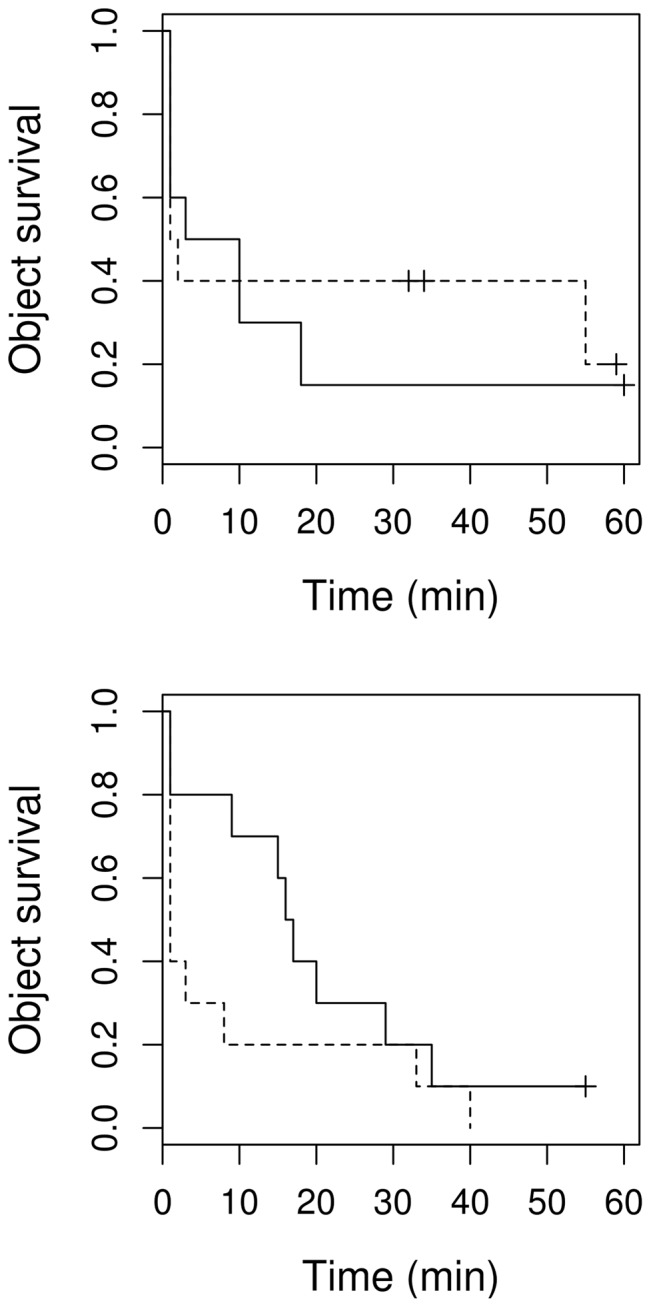
Shape dependent object survival in the nest. Survival curves of experimental objects varying in shape (dashed line - “square”, solid line - “normal egg”) in a nest during egg laying (upper) and incubation (lower). Vertical tick-marks indicate censored data.

**Figure 8:**
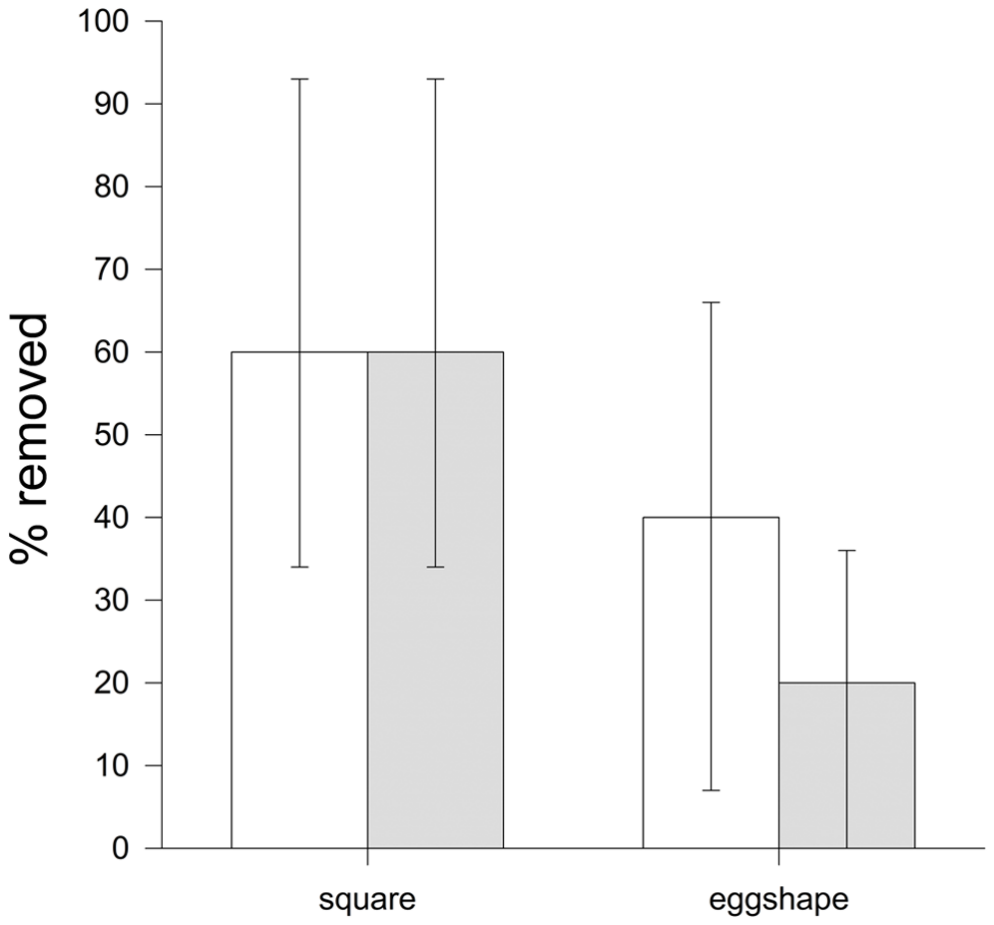
Immediate rejection in relation to object shape. Mean ± SE proportion of objects rejected in the first two min for objects varying in shape during egg laying (white bars) and incubation (grey bars). Results are expressed as the difference in rejection rate in comparison to the “normal egg” (normal egg represents the zero line).

Removal behaviour towards “square” in contrast to “normal egg” objects did not differ significantly (*df* = 1, *P* = 0.576). Similarly, there was no difference between egg laying and incubation (*df* = 1, *P* = 0.464). However, the interaction between the object and stage was almost significant (*P* = 0.096; Figure 7). Examining only the removal rate immediately after the first 2 min in relation to shape differences, we found no significant variation at all (*P*>0.6 for all; Figure 8).

## Discussion

Our results reveal that in our tree sparrow population several prerequisites in response to conspecific brood parasitism do exist. First, we found a very high interclutch variation in egg colouration but also in egg size. Using colour and size we were in fact able to significantly predict clutch affiliation for each egg, except 19 eggs (11%) of 15 nests. High interclutch variation in colour, are suggested to evolve as a result of high brood parasitism [48], [49], and may be an adaptation to increase the ability to recognise parasitic eggs in particular [50], [51]. The 19 (11%) mismatching eggs could in principle be parasitic eggs but we have no genetic proof. Second, our experiments further revealed that tree sparrows show direct anti-parasite behaviours [12]–[14], [52] but only in 37% of experimentally parasitized clutches. Third, tree sparrows are able to recognise conspecific eggs in their own nests, since only experimentally-deposited eggs have been removed. Individual recognition of parasitic eggs has been also demonstrated by other studies according to egg patterns [53], according to colour, UV reflectance [53]–[55] and egg shape [33], [54], [56].

With this background, the question arises: Why is anti-parasite behaviour not observed more frequently? Traditionally, explanations propose that rejection behaviour is influenced by the costs of parasitism relative to the costs of anti-parasite defence [19]. The costs of being parasitized by conspecifics, whereby the hosts own chicks are raised along with the parasite, are lower in comparison to interspecific brood parasitism. In the case of the common cuckoo, host reproductive success is virtually zero [57]. However, costs due to conspecific brood parasitism, like misdirected parental care, are still high enough to evolve anti-parasite defence [58]. Costs are often connected to enlarged clutch size [54] but our data do not provide evidence that a larger brood size necessarily has a negative effect on hatching success or chick body condition. E.g. we did not find any evidence for a negative effect of clutch size on hatching success (Spearman rank correlation, *r*_s_ = −0.081, *N* = 89, *P* = 0.449) or chick body mass on day 5 (hatching day as day 1) (Spearman rank correlation, *r*_s_ = 0.253, *N* = 47, *P* = 0.086).

However, such a negative effect was documented for experimentally-enlarged clutches in terms of reduced hatching success [59]–[61].

Another cost in relation to anti-parasite defence might be related to recognition errors [12], [32], when own egg(s) are rejected with any harm done to the parasitic egg [62] However, we did not detect any recognition error in relation to egg ejection during our experiment in contrast to 44% detected in house sparrow [63]. Another cost is clutch desertion, but we parasitized clutches at times when just 2 eggs were laid and the majority of pairs reacted immediately, so the costs of desertion were lower than they would have been after clutch completion [18]. Therefore, in our tree sparrows, the cost of parasitation as well as the cost of anti-parasite defence seems to be low. Thus, one key factor for developing anti-parasite behaviour in tree sparrows might be the parasitism rate and the degree of parasitism (one or more parasitic eggs).

One alternative explanation, which may contribute to explain the frequently observed discrepancy between parasitic attempts and ejection rate, is related to the motivation to eject foreign objects (including parasitic eggs) from the nest. The fact that rejection rate of our flat-objects was considerably higher than real egg ejection (see results), suggests egg ejection in tree sparrows and probably more general in small passerines, to be limited by elevated costs associated with ejection of eggs with their beaks and recognition error. If individuals are able to grasp the “egg” easily, they eject almost always (see results). However in the real world, they only eject approximately 10% of parasitic eggs. One interpretation of our results hence could be that motivation may be constraint by certain circumstances e.g. in our case the real motivation may be obscured by the ability of egg rejection and/or recognition. However, to really accurately identify the role of motivation in our results it would be also important to know how effective hosts are in identifying parasitic eggs.

In contrast, it might be also variation in motivation which may contribute to explain such a discrepancy between parasitation and ejection rate [22]. In line with this, the evolutionary background of nest sanitation behaviour might be important. Guigueno and Sealy [24] recently proposed nest sanitation to be a prerequisite for the evolution of anti-parasite defence in terms of egg ejection [25]–[27]. Our results show an almost significant increase in overall ejection rate from egg laying to incubation. We expect nest sanitation behaviour to increase at the end of incubation, because passerines remove egg shells and also start to remove chick faecal sacs immediately after hatching [24]. Thus our results are in line with the prediction namely that motivation for object ejection increases with the need for nest sanitation which should probably peak when nestlings appear. However, other studies which tested nest sanitation in these two phases did not detect a difference in rejection rate [26], [56].

Alternatively, if anti-parasite behaviour would be the driving force, one may predict a peak in ejection behaviour during the egg laying period [41], [43]. There is in fact evidence that some hosts of interspecific brood parasites reject more parasitic eggs during egg laying than later after the beginning of incubation. If hosts are parasitized later, e.g. during incubation, parasites will not hatch successfully [41], [43]. However, object appearance might play an additional role for the motivation to eject a foreign object. A simple mechanistic rule which could be applied is that motivation increases with increasing dissimilarity. However, such a mechanism is not really confirmed by our results since objects which seem most obviously different as completely white or square objects are not rejected significantly more often.

Another rule could be that motivation for object ejection increases with stimulation, which could be related to size or colour intensity. That is supported by our experiments which reveal that size is an important feature determining object ejection, as larger objects were more frequently removed. The suggestion that egg size is an important trigger of egg rejection behaviour in context to interspecific brood parasitism was also found in other studies [12], [21], [31], [32] and size might be even a stronger stimulus than colouration in some species [32].

However, motivation rules may also depend on the period. More similar objects may be discarded when anti-parasite motivation is involved. Our results showed that during egg-laying, bigger objects were not only more frequently removed, but were also more rapidly removed than smaller objects. However, this result cannot simply be explained by better visibility and hence detection of bigger objects in a nest, because the effect of size disappeared during incubation. Experimental objects were also removed during incubation, but independent of object size. Based on this result, we suggest that the motivation to reject objects which are more visible is higher during egg laying. A particular need of nest sanitation is not obvious for the egg laying stage. Thus, this sensitivity peak in discriminating objects during the egg laying stage might rather have to do with the likelihood of brood parasitism. In fact, nest sanitation experiments on yellow warblers (*Setophaga petechia*), the hosts of brown-headed cowbirds (*Molothrus ater*), revealed a similar result. In those experiments, object shape influenced rejection in pre-incubation but not during the incubation stage [64]. However, in the previous experiment, bigger objects were used. Consequently, yellow warblers were not able to remove them for several days and their main response was desertion. Our experimental objects were flat and made of hard paper, thus making them easy to remove and not causing desertion.

A closer look, however, revealed that colour variables contribute much more to differentiating between eggs of different females than size did. Differences in egg size between clutches may possibly reflect differences in female quality or condition [65], [66]. A similar change in motivation with the breeding stage may hold also for colour but surprisingly and in contrast to size, objects of different colour were removed similarly during the egg laying stage but during incubation “white egg” objects were removed much faster than “normal egg” and “dark egg” objects. The colour resemblance between the experimental object and the egg colour of a given clutch did not affect the ejection rate. The significant interaction in fact between colour and stage suggests that sensitivity regarding egg ejection changes from dark to white (see results).

We believe that the stronger reaction to white objects during incubation might be explained by the increasing necessity of egg shell removal. Egg shell removal behaviour might have become an adaptive behaviour during the late incubation stage (hatching stage) and may prevent egg capping, which is a serious danger for the hatching success of an egg [67]. Although most birds remove empty shells rapidly from the nest, there is still evidence for the occurrence of egg capping under natural conditions [68]. Tinbergen and colleagues [69] found both white and natural background colours of eggs, which resemble pieces of egg shell, to be strong stimuli for object removal in the black-headed gull (*Chroicocephalus ridibundus*). An alternative functional explanation could be the increasing need for faeces removal. Parents usually remove the white faeces sacks of their nestlings but the responsible behavioural repertoire may already develop before they hatch. In line with this possible change in motivation is also the tendency to remove squares during incubation faster than during the laying stage.

In the literature, there is almost no information about sexual differences in nest sanitation behaviour before hatching. In great reed warblers and yellow warblers it is thought that only females are responsible for burying objects because they exclusively build the nest [24]. At least for egg rejection in relation to interspecific brood parasitism it was found that in species where only females incubate, only the female was responsible for egg rejection, whereas in species where both sexes incubate, both ejected parasitic eggs [70]. In line with this our results show that both sexes removed experimental objects and both sexes participate in nest building and males regularly visit the nest box during egg laying and cover eggs during incubation [34]. Soler and colleagues [70] suggests that even in species where both sexes recognize and eject parasitic eggs females are more effective at recognition and ejection. Our data suggest that this difference might lay in the different motivation of male and female to perform anti-parasite behaviour as our experimental objects were easy to remove and strikingly different from tree sparrow eggs and still males removed significantly less experimental objects than female.

In conclusion, our tree sparrow population shows high interclutch colour variation, which often evolves under the pressure of brood parasites. The presence of anti-parasite behaviour supports our suggestion that also brood parasitism maybe responsible for variation in egg features, as we have found that they can recognise and reject conspecific eggs in their clutch. Moreover, experiments with different objects revealed that the motivation of tree sparrows to remove experimental objects from their nests was highest during egg laying for objects of varying size, most likely because of parasitism risk at this breeding stage. In contrast, during incubation, the motivation was higher to remove white objects and objects with edges. That might be driven by the necessity to remove shells and/or faecal sacks after hatching. We suggest that the frequently observed discrepancy between parasitism rate and ejection rate is related to the motivation to eject foreign objects from the nest. The fact that rejection rate of our flat-objects was considerably higher than real egg ejection, suggests egg ejection in small passerines, to be limited by elevated costs to eject eggs with their beaks. Overall, it seems that in tree sparrows the nest sanitation plays a key role in the evolution of the removal of parasitic eggs.
